# Depletion of mmu_circ_0001751 (circular RNA Carm1) protects against acute cerebral infarction injuries by binding with microRNA-3098-3p to regulate acyl-CoA synthetase long-chain family member 4

**DOI:** 10.1080/21655979.2022.2032971

**Published:** 2022-02-03

**Authors:** Rui Mao, Hua Liu

**Affiliations:** Department of Neurology, The Third People’s Hospital of Chengdu, Chengdu City, Sichuan Province, China

**Keywords:** Acute cerebral infarction, ferroptosis, circ-Carm1, miR-3098-3p, ACSL4

## Abstract

Circular RNAs (circRNAs) play a critical role in acute cerebral infarction (ACI). Our research discussed the effect of circ-Carm1 in ACI and its potential molecular mechanisms. Healthy controls and patients with ACI were included in this study. The establishment of an oxygen and glucose deprivation/reoxygenation (OGD/R) model of HT22 cells was conducted to mimic ACI *in vitro*. Quantitative reverse transcription polymerase chain reaction was conducted to determine mRNA levels extracted from serum and HT22 cell samples, and Western blotting was performed to determine protein levels. Terminal deoxynucleotidyl transferase dUTP nick end labeling and cell counting kit 8 assays were conducted to evaluate cellular functions. Concentrations of Fe^2+^ and malondialdehyde, and levels of transferrin receptor 1, glutathione peroxidase 4, and glutathione were evaluated to determine ferroptosis in OGD/R-induced HT22 cells. The binding relationships between mRNAs and miRNAs were verified. circ-Carm1 was highly expressed in OGD/R-treated HT22 cells. Deficiency of circ-Carm1 restored cell viability and suppressed ferroptosis in OGD/R-induced HT22 cells. miR-3098-3p was predicted to be a target of circ-Carm1. The miR-3098-3p inhibitor partly neutralized the functions of circ-Carm1 in OGD/R-induced HT22 cells. Furthermore, acyl-CoA synthetase long-chain family member 4 (ACSL4) was confirmed to be a downstream target of miR-3098-3p and was elevated in OGD/R-induced HT22 cells. Overexpression of ACSL4 mitigated the functions of miR-3098-3p and accelerated HT22 cell dysfunction. Hence, circ-Carm1 is upregulated in ACI. circ-Carm1 suppression protects HT22 cells from dysfunction by inhibiting ferroptosis. Therefore, inducing circ-Carm1 deficiency may be a promising therapeutic method for ACI.

## Graphical Abstract

**Figure:**
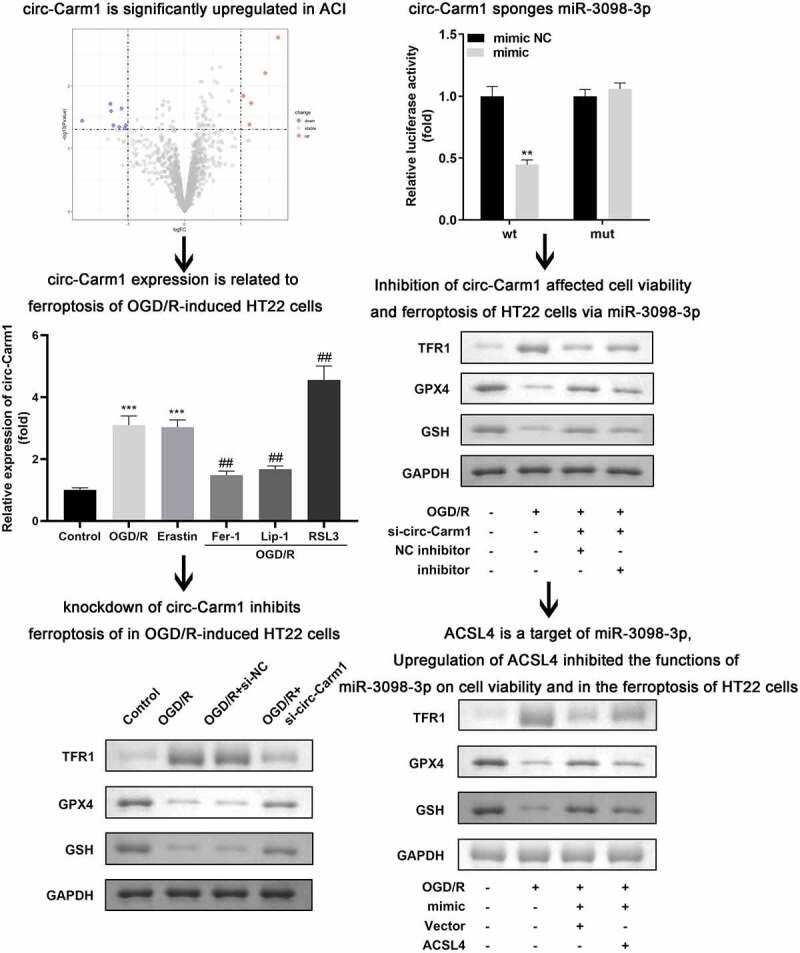

## Introduction

Acute cerebral infarction (ACI) is an ischemic cerebrovascular disease that seriously endangers human health, and its prevalence and disability rates are increasing [1]. In the process of ACI, ischemia causes irreversible damage to brain cells, and the focus of infarction with poor vascular compensation expands rapidly, thereby causing serious complications [1]. Hence, timely diagnosis and effective treatment are vital in treating patients with ACI as shown in Table 1.

**Table 1.** General characteristics of the ACI patients

Parameter	Circ-carm1			miR-3098-3p			ACSL4		
	Low (n = 13)	High (n = 19)	*p*-value	Low (n = 22)	High (n = 10)	*p*-value	Low (n = 15)	High (n = 17)	*p*-value
Age,years	47.92 ± 5.27	46.89 ± 4.31	0.5490	48.14±4.85	45.50±3.84	0.1410	46.20 ± 3.88	48.29 ± 5.18	0.2104
BMI, kg/m^2^	23.00 ± 2.06	23.65 ± 1.41	0.2938	23.08±1.73	24.06±1.51	0.1369	23.89 ± 2.11	22.94 ± 1.14	0.1172
SBP, mmHg	142.20 ± 4.88	138.89 ± 10.12	0.2839	142.51 ± 7.68	137.44 ± 9.78	0.2117	139.46 ± 8.33	140.92 ± 8.75	0.6326
DBP, mmHg	85.63 ± 6.10	86.70 ± 10.44	0.7440	86.67 ± 7.87	85.37 ± 11.08	0.7063	83.87 ± 9.63	88.37 ± 7.74	0.1533
Creatinine (Cr), μmol/L	84.59 ± 11.27	85.28 ± 8.50	0.8452	82.92 ± 7.58	89.58 ± 12.10	0.0666	84.48 ± 8.84	85.46 ± 10.38	0.7763
HDL-C, mmol/L	1.46 ± 0.36	1.31 ± 0.46	0.3317	1.38 ± 0.39	1.35 ± 0.52	0.8708	1.51 ± 0.28	1.24 ± 0.50	0.0808
LDL-C, mmol/L	2.62 ± 0.38	2.86 ± 0.43	0.1167	2.67 ± 0.37	2.97 ± 0.48	0.0623	2.82 ± 0.45	2.71 ± 0.40	0.4717
Triglyceride (TG), mmol/L	1.82 ± 0.48	2.04 ± 0.23	0.0881	1.91 ± 0.4	2.05 ± 0.27	0.3265	1.97 ± 0.45	1.94 ± 0.28	0.8389
Total cholesterol (TC), mmol/L	5.27 ± 0.88	5.05 ± 0.50	0.3849	4.99 ± 0.59	5.47 ± 0.78	0.0653	5.22 ± 0.71	5.07 ± 0.66	0.5329

In recent years, serological markers, including bilirubin, alkaline phosphatase, uric acid, low-density lipoprotein cholesterol, and high-density lipoprotein cholesterol have been shown to be associated with ACI [2–5]. However, clinical results have found that the risk of ACI still cannot be accurately predicted in a large number of people, suggesting that these indicators do not play a significant role in the clinical value of early prediction and diagnosis of ACI.

For the past few years, an increasing number of studies have found that although non-coding RNAs (ncRNAs) cannot directly encode proteins, they can participate in biochemical processes such as RNA transcription and protein expression in several ways [6]. Circular RNA (circRNA) is a novel ncRNA that differs from linear RNA and is characterized by a covalently closed-loop and erase-resistant degradation. This special structure makes circRNAs more stable than other ncRNAs [7,8]. CircRNA may be involved in the post-transcriptional regulation of human disease-related genes and is regarded as a sponge microRNA (miRNA) [8]. In recent years, RNA high-throughput sequencing has been widely used to provide strong evidence for the involvement of circRNAs in the pathological process of ACI [9,10]. For instance, Hu XL et al, applied high-throughput sequencing techniques to identify the differential changes of plasma circRNAs expression in patients with ACI [11]. circHECTD1 was identified by analyzing circRNA profiling, and research data by Han B demonstrated that circHECTD1 can function as an early indicator and therapeutic target for stroke [12]. Although the biological importance of circRNA is established, little is known about its expression and biological function in the pathogenesis of ACI.

In the current study, we aimed to identify an aberrantly expressed circRNA of ACI using circRNA microarray GSE115697. Then, the cellular function and mechanism of selected circRNAs were investigated to provide a basis for finding new markers of ACI.

## Materials and methods

### Reagent

Ferroptosis agonists Erastin (S7242, 4.6 μM) and RSL3 (SIM; S8155, 0.5 µM), and ferroptosis inhibitors ferrostatin-1 (Fer-1; S7243, 5 μM) and liproxstatin-1 (Lip-1; S7699, 22 μM) were purchased from Selleck (Shanghai, China).

### Bioinformatics

A microarray profile GSE115697 related to ACI was obtained from the Gene Expression Omnibus database. All differentially expressed circRNAs were identified and screened under the standard *P* < 0.05 and |log_2+_FC| ≥ 1.0. In addition, Starbase v.3.0 and TargetScan v.7.2 were used to predict the binding sites between mRNA and miRNA.

### Object of study

A total of 32 patients with ACI, who were hospitalized in the Department of Neurology, The Third People’s Hospital of Chengdu from July 2019 to June 2020 were enrolled, and 32 healthy volunteers who underwent a physical examination at our hospital were included in the control group. Venous blood (4 mL) was taken from the median cubital vein of the patients 12 h after fasting. The upper serum was extracted after centrifugation (3000 rpm, 10 min) and stored at −80°C for further processing.

Inclusion criteria:

1. Patients who met the diagnostic criteria of ACI in the Chinese Guidelines for the Diagnosis and Treatment of Acute Ischemic Stroke (2018 edition).

2. Patients with initial episode of ACI and those with an onset within 72 h.

3. Patients and their family members agreed to the experiment and signed a relevant informed consent.

Exclusion criteria:

1. Patients with ACI complicated by intracranial hematoma, cerebral hemorrhage, cerebrovascular malformation, intracranial tumor, cerebral amyloidosis, and simple cerebral lobe microhemorrhage. Likewise, those with mental illness, severe cardiopulmonary dysfunction, severe liver insufficiency, and systemic infections.

2. Patients with a previous history of stroke, malignancy, and infectious diseases; presence of any vascular disease, concomitant serious heart, liver and kidney diseases, hematological diseases, central sclerosis, and central nervous system infection.

### Establishment of an oxygen and glucose deprivation/reoxygenation (OGD/R) model

A mouse hippocampal neuronal cell line (HT22) purchased from public cell banks (ATCC, USA) was used to mimic ACI *in vitro*. Briefly, HT22 cells were cultivated in glucose-free and serum-free DMEM (Sigma-Aldrich, USA) under 5% CO_2_ and 95% N_2_ for 30 min. HT22 cells were then cultivated under standard culture conditions for 24 h [13].

### Cell transfection

si-circ-Carm1 1#, si-circ-Carm1 2#, miR-3098-3p mimic/inhibitor, ACSL4, and their negative control plasmids (Abiocenter Biotech, USA) were transfected into HT22 cells with Lipofectamine® 3000 reagent (Invitrogen, USA) following the manufacturer’s instructions.

### Quantitative reverse transcription polymerase chain reaction (RT-qPCR)

The total RNA of serum or HT22 cells from each group was extracted using a Trizol reagent (Thermo Fisher Scientific, USA) according to the manufacturer’s instructions. Then, the concentrations of extracted RNA were evaluated at 260 nm and 280 nm using a Nanodrop 2000 spectrophotometer (Thermo Fisher Scientific, USA). To obtain cDNA, miRNA polyadenylation and reverse transcription were performed using the mir-x^TM^miRNA First-chain Synthesis Kit (Takara, China). Similarly, mRNA and circRNA polyadenylation and reverse transcription were performed using PrimeScript^TM^RT Master Mix from Takara. RNA was quantified using SYBR Green Realtime PCR Master Mix (Toyobo, Japan) and Q6 QuantStudio^TM^ 12 K Flex real-time PCR System (Thermo Fischer Scientific, USA). Fluorescence was quantified with initial activation at 95°C for 10 min, followed by 40 cycles of denaturation at 94°C for 15s, annealing at 55°C for 30s, and extension at 70°C for 30s. Data were analyzed using the 2^−ΔΔCt^ method [14]. All primers were purchased from Shanghai GenePharma Co., Ltd.

### Cell counting kit 8 (CCK-8)

Cell viability was determined using a CCK-8 kit [15]. One hundred microliters per well of dense medium containing 1 × 10^5^ cells/mL HT22 cells were placed into 96-well plates. The CCK-8 reagent (AMJ-KT0001; AmyJet Technology, China) was added to each well, and HT22 cells were cultivated in an incubator at 37°C for 4 h to measure cell viability. Absorbance was measured at a wavelength of 450 nm.

### Terminal deoxynucleotidyl transferase dUTP nick end labeling (TUNEL) assay

Cell death of HT22 cells in each group was evaluated using the One-step TUNEL Cell Apoptosis Detection Kit (Beyotime Biotech, China). The cells were washed with PBS twice. Then, 50-μL TUNEL detection solution was added to the cells before being incubated at 3°C in the dark for 1 h. After which, the cells were suspended in 500 μL PBS, which were observed under a fluorescence microscope in an excitation wavelength range of 460 nm (green channel) and emission wavelength range of 565 nm (blue channel).

### Determination of Fe^2+^ and malondialdehyde (MDA) concentration

Labile iron exists mainly in the ferrous (Fe^2+^) form [16]. The Fe^2+^ levels were measured using an iron colorimetric assay kit (ScienCell, USA), according to the manufacturer’s instructions. Briefly, HT22 cells treated with an iron assay buffer were centrifuged at 13,000 × g for 10 min at 4°C, and 50 μL of the supernatant was cultivated with 50 μL of buffer for 30 min at 25°C. After 200 μL of reagent mix was added to the mixture in the dark for 30 min at 25°C, we measured the absorbance at 593 nm using a microplate reader. The levels of MDA in HT22 cells were detected following the manufacturer’s instructions of a commercial kit (MAK085, Sigma-Aldrich, USA).

### Western blot assay

RIPA reagents (Sigma-Aldrich, USA) were used to extract proteins from HT22 cells. The protein concentration was evaluated using a BCA kit (Sigma-Aldrich, USA). Eligible proteins were isolated by 15% SDS-PAGE gel and then transferred onto PVDF membranes (Millipore, USA), which were blocked with 5% defatted milk for 2 h. Then, the membranes were incubated with primary antibodies (Abcam, USA), including transferrin receptor 1 protein (TFR1; ab214039, 1: 1000), glutathione peroxidase 4 (GPX4; ab125066, 1: 1000), glutathione (GSH; ab261738, 1: 1000), and mouse anti-GAPDH (ab9485, 1: 500) at 4°C overnight, followed by incubation with secondary goat anti-mouse antibody to immunoglobulin G (IgG; ab205719, 1: 2000) and goat anti-rabbit antibody to IgG (ab6721, 1:2000) for 1 h. Protein expression was determined using an ECL kit (ab133406, Abcam, USA). Finally, the protein bands were visualized using an enhanced chemiluminescence system (Thermo Fisher Scientific, USA). GAPDH was used as the loading control.

### Dual luciferase reporter gene assay

The wild-type (wt) and mutant (mut) 3-UTR regions of circ-Carm1 and ACSL4 luciferase reporter vectors were purchased from Guangzhou RiboBio Co., Ltd. (Guangzhou, China). HT22 cells were transfected with miR-3098-3p mimic or miR-NC mimic and the wt or mut of circ-Carm1 or ACSL4 for 48  h. The cells were then lysed to measure luciferase activity using a luciferase reporter assay kit (RG027; Beyotime, China). Firefly luciferase activity was normalized to Renilla luciferase activity.

### RNA pull-down assay

RNA pull-down assays were performed using the MagCapture^TM^ RNA Pull-Down Assay Kit (297–77,501; Whatman, UK) [17]. First, synthetic biotin-labeled RNA (Biotin-miR-NC and Biotin-miR-3098-3p) were incubated with the cell lysate, and streptavidin-labeled magnetic beads were resuspended to capture target RNA at 4°C overnight. Finally, the magnetic beads were eluted from the protein complexes. The results were determined using RT-qPCR.

Fluorescence in situ hybridization

After digestion, HT22 cells were incubated at 37°C for 1 h with hybridization fluid of circRNA-Probe (8 ng/μL). After hybridization, the cells were washed, followed by adding DAPI dye solution, and incubated at dark for 8 min. After rinsing, anti-fluorescence quenching agent was dropped [18]. Images were observed and collected under nikon ECLIPSE CI fluorescence microscope (Tokyo, Japan). Each experiment was repeated three times.

### Statistical analysis

Data were analyzed using SPSS version 19.0 and presented as $\overset{ˉ}{x}\pm SD$. Statistical significance of the differences was evaluated using Student’s *t*-test for two groups, and one-way analysis of variance (one-way ANOVA) followed by Tukey’s post hoc test for multiple groups. Differences were considered statistically significant at p < 0.05. All experiments were performed in triplicate.

## Results

### Abnormally high circ-Carm1 expression in OGD/R-induced HT22 cells with ferroptosis

**Figure 1.**
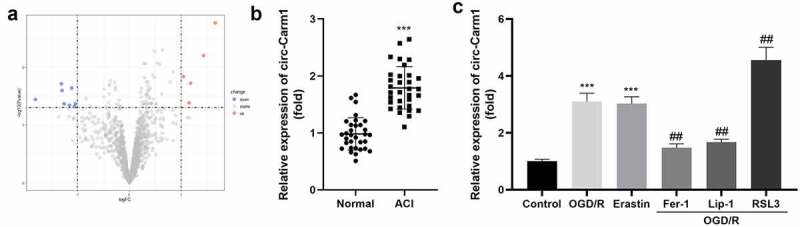
Circ-Carm1 is highly expressed in the ACI. (a) Volcano map of dysregulated circRNAs identified in ACI. (b) circ-Carm1 expression in the serum of patients with ACI and healthy volunteers was measured by RT-qPCR. (c) circ-Carm1 expression in HT22 cells treated with OGD/R performance, 4.6 μM Erastin, 5 μM ferrostatin-1 (Fer-1), 22 μM liproxstatin-1 (Lip-1), and 0.5 µM RSL3 separately was detected by RT-qPCR.

Firstly, dysregulated circRNA in ACI were identified from bioinformatic analyses. As indicated in Figure 1a, eight aberrantly downregulated and five abnormally upregulated circRNAs were identified, among which, circ-Carm1 was the most significantly upregulated circRNA (Figure 1(a)). The mRNA levels of circ-Carm1 in the serum of patients with ACI were significantly higher than those of the healthy group (Figure 1(b)). Moreover, circ-Carm1 was found to be dramatically increased in HT22 cells after OGD/R treatment. An increase in circ-Carm1 was also induced by erastin, a ferroptosis activator. Furthermore, Fer-1 and Lip-1, two ferroptosis inhibitors, decreased circ-Carm1 expression in OGD/R-induced HT22 cells, while RSL3 further upregulated circ-Carm1 expression in the OGD/R group (Figure 1(c)).

***P < 0.001, vs. normal and control group. ^##^*P* < 0.01, vs. OGD/R group.

### Knockdown of circ-Carm1 restored cell viability and inhibited ferroptosis in OGD/R-induced HT22 cells

**Figure 2.**
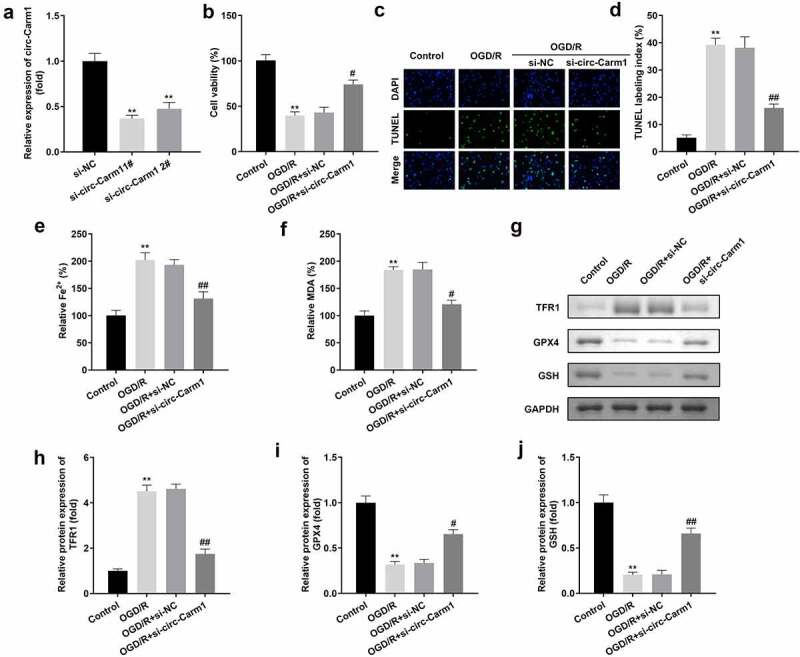
Depletion of circ-Carm1 restores cell viability and inhibits ferroptosis of HT22 cells. (a) circ-Carm1 expression levels were determined by RT-qPCR after circ-Carm1 knockdown. (b) Cell viability of HT22 cells was detected using the CCK-8 assay before and after circ-Carm1 knockdown. (c-d) TUNEL staining was used to detect cell death before and after circ-Carm1 knockdown. (e-f) Levels of Fe^2+^ and malondialdehyde (MDA) were determined using an enzyme-linked immunosorbent assay. (g-j) Protein levels of transferrin receptor 1 (TFR1), glutathione peroxidase 4 (GPX4), and glutathione (GSH) were detected by Western blotting.

Then, the effect of circ-Carm1 on OGD/R-induced HT22 cells was evaluated. circ-Carm1 was successfully inhibited by a siRNA specific for circ-Carm1, which was more potent in the si-circ-Carm1 1# group (Figure 2(a)). Deficiency of circ-Carm1 restored cell viability (Figure 2(b)) and inhibited cell death, which was promoted in the OGD/R group (Figures 2(c-d)). Simultaneously, the increased secretion of Fe^2+^ and MDA in the OGD/R group was dramatically ameliorated by knockdown of circ-Carm1 (Figures 2(e-f)). Additionally, the protein concentration of ferroptosis-related proteins, including TFR1, GPX4, and GSH, was detected in HT22cells, and the results indicated that an increase in TFR1 and a decrease in GPX4 along with GSH induced by OGD/R performance were reversed by the depletion of circ-Carm1 (Figure 2(g-j)).

**P < 0.01, vs. si-NC and control group. ^#^*P* < 0.05, ^##^*P* < 0.01, vs. OGD/R group.

### Circ-Carm1 sponges miR-3098-3p

**Figure 3.**
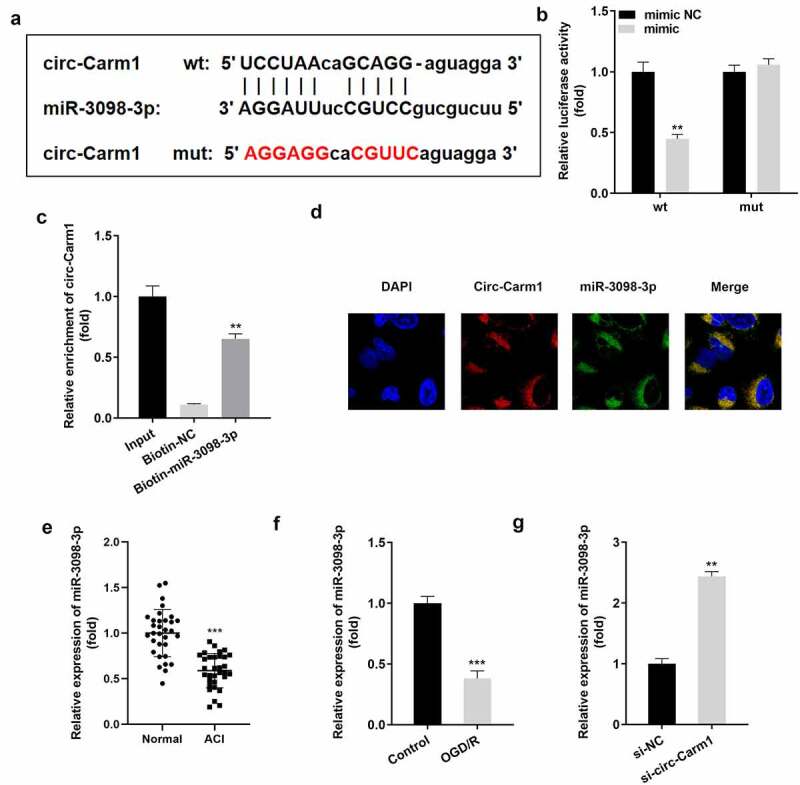
Carm1 sponges miR-3098-3p. (a) The binding sites between miR-3098-3p and circ-Carm1 were predicted by bioinformatics analysis. (b) Relative luciferase activity of HT22 cells co-transfected with wt circ-Cram1 and miR-3098-3p mimics. (c) RT-qPCR analysis of circ-Carm1 expression enriched in biotin containing miR-3098-3p. (d) The FISH assay was used to detect the location of circ-Carm1 and miR-3098-3p. (e) miR-3098-3p expression in the serum of patients with ACI and healthy volunteers was measured by RT-qPCR. (f) RT-qPCR analysis of miR-3098-3p expression in OGD/R-induced HT22 cells. (g) RT-qPCR analyses of miR-3098-3p expression in HT22 cells transfected with si-circ-Carm1.

Next, binding relationship between circ-Carm1 and its target miRNA was studied. Figure 3(a) illustrates the binding sites predicted by StarBase between circ-Carm1 and miR-3098-3p. The luciferase activity of HT22 cells co-transfected with a luciferase-labeled miR-3098-3p mimic along with wt circ-Carm1 plasmids was lower than that in the negative control group, while there was no significant difference in the mut groups (Figure 3(b)). The consequence of the RNA pull-down assay revealed that circ-Carm1 was more notably enriched in the Biotin-miR-3098-3p group than in the Biotin-NC group (Figure 3(c)). Furthermore, circ-Carm1 and miR-3098-3p were located in cytoplasm (Figure 3(d)). miR-3098-3p was expressed at lower levels in ACI patients (Figure 3(e)) and OGD/R-treated HT22 cells (Figure 3(f)) as compared to that in control group, but was upregulated by the knockdown of circ-Carm1 (Figure 3(g)).

**P < 0.01, ***P < 0.001, vs. mimic NC, Biotin-NC, normal, control, and si-NC group.

### Inhibition of circ-Carm1 affected cell viability and ferroptosis of HT22 cells via miR-3098-3p

**Figure 4.**
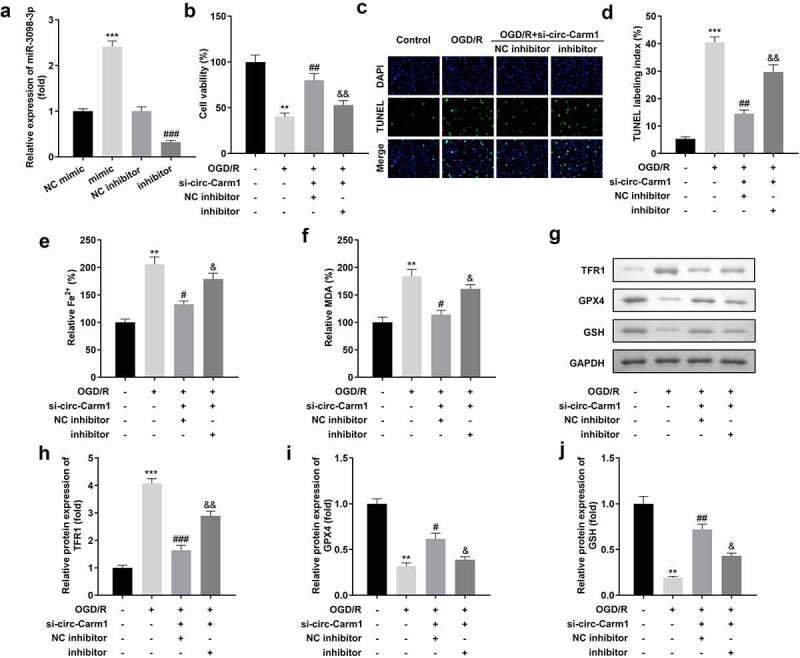
Downregulation of miR-3098-3p reverses the effects of circ-Carm1 deficiency on cell viability and ferroptosis of HT22 cells. (a) miR-3098-3p expression levels were detected using RT-qPCR after transfection. (b) Cell viability of HT22 cells co-transfected with si-circ-Carm1 and miR-3098-3p inhibitor and was detected using the CCK-8 assay. (c-d) TUNEL staining was conducted to detect cell death in cells co-transfected with si-circ-Carm1 and miR-3098-3p inhibitor. (e-f) Levels of Fe^2+^ and malondialdehyde (MDA) were determined using an enzyme-linked immunosorbent assay. (g-j) Protein levels of transferrin receptor 1 (TFR1), glutathione peroxidase 4 (GPX4), and glutathione (GSH) were detected by Western blotting.

Whether miR-3098-3p could affect the function of circ-Carm1 on HT22 cells were then been discussed. We successfully inhibited or overexpressed miR-3098-3p separately after HT22 cells were transfected with miR-3098-3p inhibitor or miR-3098-3p mimic, respectively (Figure 4(a)). Moreover, miR-3098-3p inhibitor suppressed si-circ-Carm1-induced effects on cell viability (Figure 4(b)) and cell death (Figures 4(c-d)). Downregulation of miR-3098-3p also reversed the regulatory roles of circ-Carm1 inhibition by promoting the secretion of Fe^2+^ and MDA (Figures 4(e-f)). Moreover, miR-3098-3p improved the effects of circ-Carm1 knockdown on the protein concentrations of TFR1, GPX4, and GSH (Figure 4(g-j)).

**P < 0.01, ***P < 0.001,vs. NC mimic and control group. ^#^*P* < 0.05, ^##^*P* < 0.01, ^###^*P* < 0.001, vs. NC inhibitor and OGD/R group. ^&^*P* < 0.05, ^&&^*P* < 0.01, vs. OGD/R + si-circ-Carm1 + NC inhibitor group.

### ACSL4 is a target of miR-3098-3p

**Figure 5.**
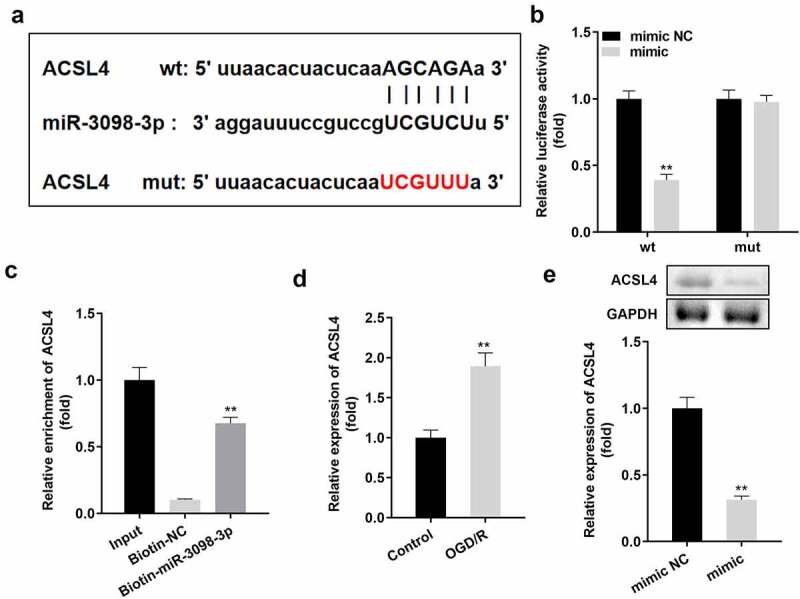
ACSL4 is a target gene of miR-3098-3p. (a) Binding sites between miR-3098-3p and ACSL4 were predicted by bioinformatics analysis. (b) Relative luciferase activity of HT22 cells co-transfected with wt ACSL4 and the miR-3098-3p mimic. (c) RT-qPCR analyses of ACSL4 expression enriched in biotin containing miR-3098-3p. (d) RT-qPCR analysis of ACSL4 expression in OGD/R-induced HT22 cells. (e) ACSL4 protein and mRNA expression in HT22 cells transfected with the miR-3098-3p mimic.

Figure 5(a) indicates that ACSL4 is a downstream target gene of miR-3098-3p. Dual luciferase reporter and RNA pull-down assays confirmed the interaction between ACSL4 and miR-3098-3p (Figures 5(b-c)). Additionally, ACSL4 expression was higher in OGD/R-treated HT22 cells than in untreated HT22 cells (Figure 5(d)) and was downregulated by miR-3098-3p overexpression on both protein and mRNA level (Figure 5(e)).

**P < 0.01, vs. mimic NC, Biotin-NC, control, and mimic NC group.

### Upregulation of ACSL4 inhibited the functions of miR-3098-3p on cell viability and in the ferroptosis of HT22 cells

**Figure 6.**
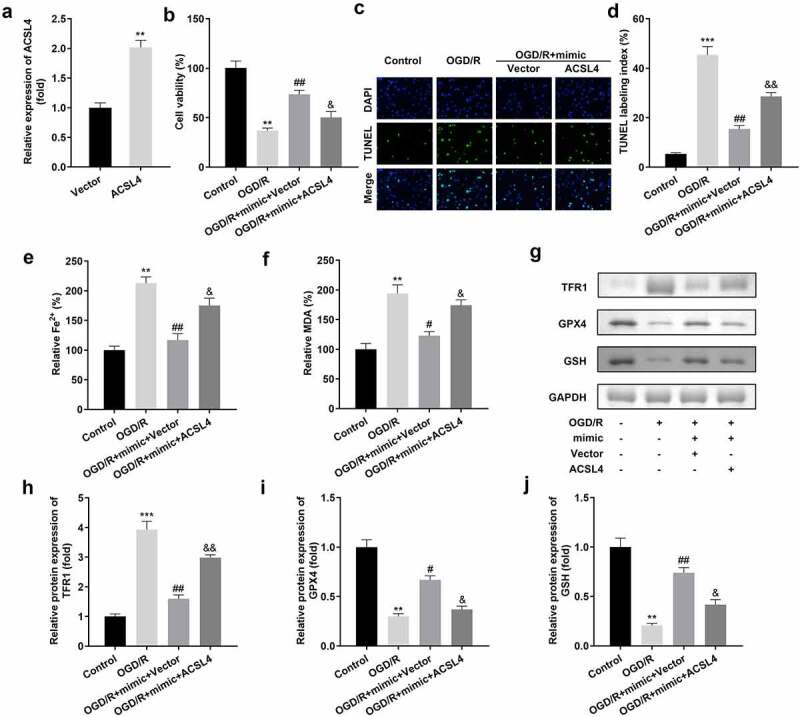
Overexpression of ACSL4 inhibits the functions of miR-3098-3p. (a) miR-3098-3p expression levels were detected using RT-qPCR after transfection. (b) Cell viability of HT22 cells co-transfected with miR-3098-3p mimic and overexpressing ACSL4 plasmids were detected using the CCK-8 assay. (c-d) TUNEL staining was used to detect cell death in cells co-transfected with the miR-3098-3p mimic and overexpressed ACSL4 plasmids. (e-f) Levels of Fe^2+^ and malondialdehyde (MDA) were determined using an enzyme-linked immunosorbent assay. (g-j) Protein levels of transferrin receptor 1 (TFR1), glutathione peroxidase 4 (GPX4), and glutathione (GSH) were detected by Western blotting.

We overexpressed ACSL4 in miR-3098-3p-upregulated HT22 cells to determine the function of ACSL4 on miR-3098-3p in ACI (Figure 6(a)). Compared with OGD/R-induced HT22 cells overexpressing miR-3098-3p, OGD/R-induced HT22 cells co-transfected with miR-3098-3p mimic along with overexpressed ACSL4 vectors inhibited cell viability (Figure 6(b)) and aggravated ferroptosis (Figure 6(c-j)).

**P < 0.01, ***P < 0.001,vs. vector and control group. ^#^*P* < 0.05, ^##^*P* < 0.01, vs. OGD/R group. ^&^*P* < 0.05, ^&&^*P* < 0.01, vs. OGD/R + mimic + vector group.

## Discussion

ACI has become a major national health hazard due to its high mortality and disability rates, and the in-depth study of circRNA provides a new approach for the treatment of cerebral infarction and ischemic reperfusion [1,12]. In the present study, we identified a novel circRNA, circ-Carm1, which was dramatically increased in ACI. Functionally, we found that circ-Carm1 was evidently abundant in ACI model cells, and knockdown of circ-Carm1 notably restored cell viability and inhibited ferroptosis in ACI model cells. Mechanistically, circ-Carm1 sponged miR-3098-3p to upregulate ACSL4 expression in ACI model cells to participate in ACI progression *in vitro*.

Iron is a double-edged sword in the dynamic balance of brain tissue; on the one hand, iron is essential for the normal brain to produce large amounts of ATP; on the other hand, the brain is highly susceptible to iron-dependent oxidative stress [19]. Previous studies have reported that the primary causes of neuronal death are necrosis, apoptosis, and autophagy, but none of these mechanisms can fully explain the early brain injury caused by acute central nervous system diseases [20,21]. Ferroptosis, a non-apoptotic form of cell death, is characterized by the accumulation of iron-dependent lipid hydroperoxides [22]. Ferroptosis is accompanied by changes in GSH, Fe2^+^, and MDA [23–25]. Accumulating evidence demonstrates that ferroptosis is becoming an important mechanism of pathological cell death during stroke and other acute brain injuries, and some studies have shown that ferroptosis inhibitors can reverse neurological damage [26–28]. For instance, Hui et al. demonstrated that compound tongluo decoction suppresses ferroptosis in ACI [29]. Electroacupuncture treatment has also been reported to improve ACI by suppressing ferroptosis [27]. Therefore, inhibition of ferroptosis is key to treating ACI. In the current study, we identified circ-Carm1 as a novel dysregulated circRNA in ACI, and circ-Carm1 expression had a positive relationship with the activation of ferroptosis in ACI model cells. These findings are in line with those of previous studies showing that ferroptosis contributes to ACI [30,31]. Our data revealed that after circ-Carm1 was knocked down in ACI model cells, cell viability was restored, and ferroptosis was inhibited. Therefore, we speculated that circ-Carm1 may affect the ACI process by regulating ferroptosis.

It is well known that all circRNAs serve as sponges for miRNAs to regulate cellular processes. Our predictive analysis and verification experiments suggested that miR-3098-3p can bind to circ-Carm1. Although miR-3098-3p has not yet been found to play a regulatory role in diseases, our data indicated that miR-3098-3p expression was downregulated in ACI model cells. In addition, inhibition of miR-3098-3p partly abrogated the effects of circ-Carm1 on cell viability and ferroptosis. circ-Carm1 deficiency may protect against ACI by regulating miR-3098-3p.

ACSL4 is an essential gene for ferroptosis sensitivity [27,32]. Downregulation of ACSL4 has been found to be a novel treatment method for ischemic stroke by suppressing ferroptosis-induced brain damage [33]. In the current study, ACSL4 was confirmed to be a downstream target gene of miR-3098-3p and was upregulated in ACI model cells. Increased ACSL4 contradicted the effect of miR-3098-3p mimic in reducing ferroptosis. ACSL4 may promote ferroptosis and aggravate the dysfunction of ACI model cells, which is consistent with the findings of Doll et al. and Cui et al. [27,34].

## Conclusions

Taken together, cir-Carm1 deficiency protected against ACI by regulating miR-3098-3p/ACSL4 *in vitro*. Therefore, the circ-Carm1/miR-3098-3p/ACSL4 axis may be a promising therapeutic strategy for ACI.

## Supplementary Material

Supplemental Material

## Data Availability

The datasets used and analyzed during the current study are available from the corresponding author on reasonable request.

## Highlights

- circ-Carm1 is highly expressed in ACI.
- circ-Carm1 depletion restores cell viability and inhibits HT22 cell ferroptosis.
- ACSL4 overexpression inhibits the functions of miR-3098-3p.
