# Supplementary figures and images for: Depletion of mmu_circ_0001751 (circular RNA Carm1) protects against acute cerebral infarction injuries by binding with microRNA-3098-3p to regulate acyl-CoA synthetase long-chain family member 4
Source: Bioengineered. 2022 Feb 3;13(2):4063–75. doi: 10.1080/21655979.2022.2032971 (PMC8974190; doi:10.1080/21655979.2022.2032971)

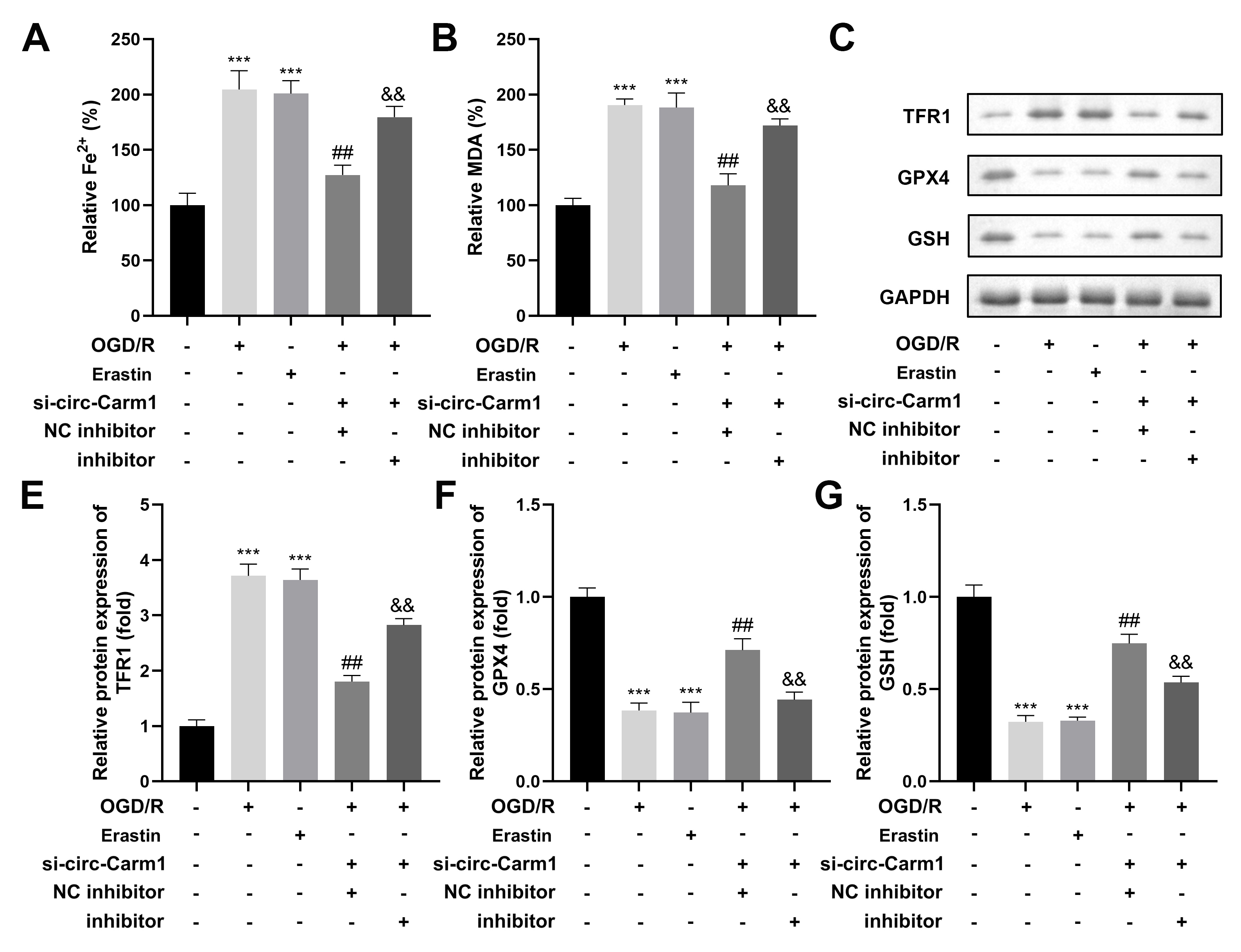

Supplement: Supplemental Material [file KBIE_A_2032971_SM5547.zip › supplementary/Supplementary material 1.jpg]

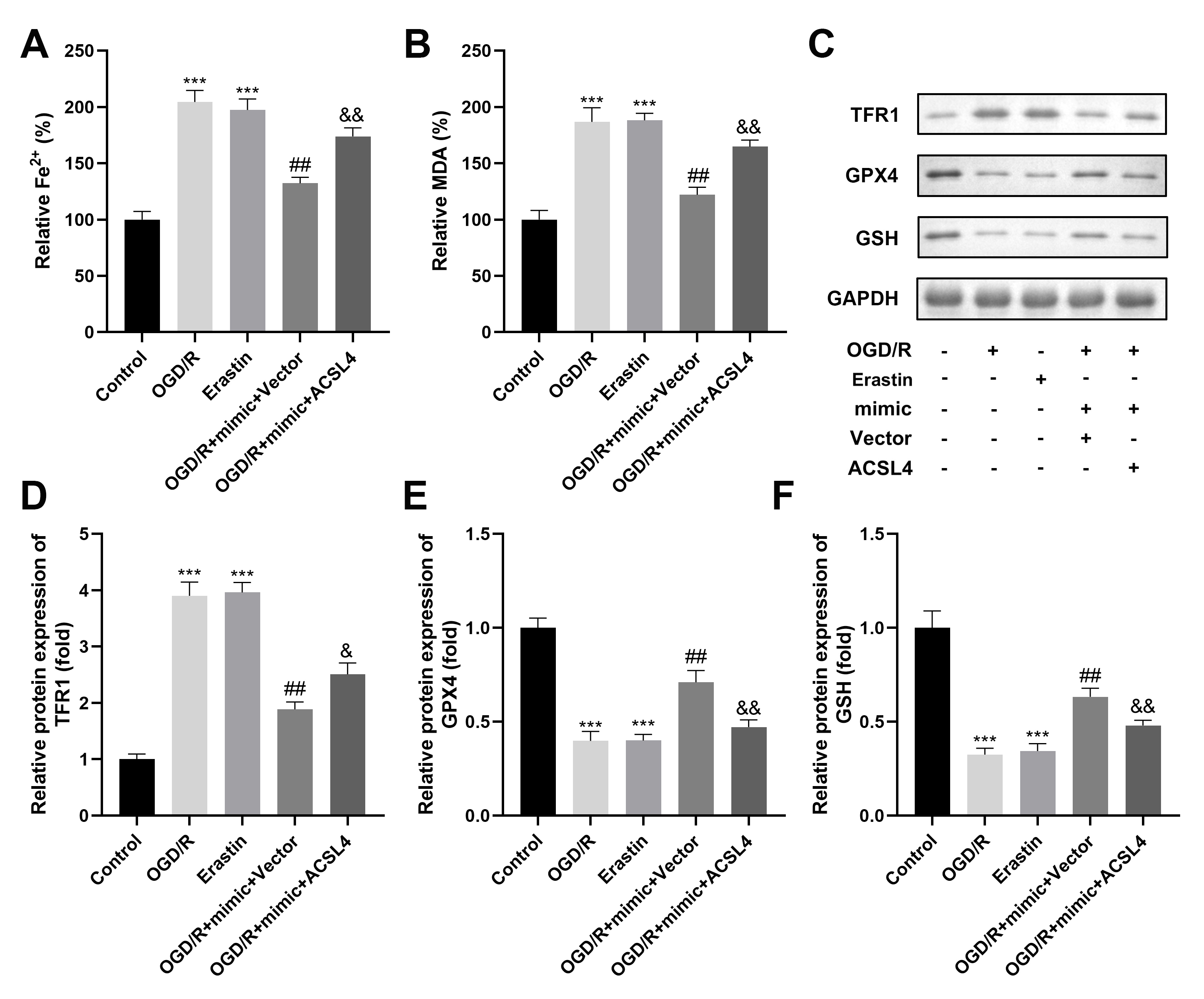

Supplement: Supplemental Material [file KBIE_A_2032971_SM5547.zip › supplementary/Supplementary material 2.jpg]
